# Cleaning and pre-processing of actigraphy data for physical activity and sleep research: a scoping review

**DOI:** 10.1088/1361-6579/ae3b96

**Published:** 2026-03-03

**Authors:** S G Gonsalves, J J Zhao, A A Livinski, M Steele, A Ross, T Fuss, K Clevenger, L N Saligan

**Affiliations:** 1 National Institute of Nursing Research, National Institutes of Health, Bethesda, MD, United States of America; 2 Duke University School of Medicine, Duke University, Durham, NC, United States of America; 3 National Institutes of Health Library, Office of Research Services, Office of the Director, National Institutes of Health, Bethesda, MD, United States of America; 4 Montgomery College, Silver Spring, MD, United States of America; 5 Utah State University, Kinesiology and Health Science, Emma Eccles Jones College of Education and Human Services, Logan, UT, United States of America; 6 Rutgers University, School of Nursing, Newark, NJ, United States of America

**Keywords:** actigraphy, physical activity, sleep, free-living activity, scoping review

## Abstract

*Objective*. Numerous studies examine the link between health and sleep-wake patterns to understand etiology, establish preventive algorithms, or develop therapeutics. The use of actigraphy to measure physical activity (PA) and sleep is increasing, partly because of its non-invasive nature and its ability to continuously monitor PA and sleep in free-living settings. There are several actigraphy data cleaning and pre-processing methods, but there is no consensus on how to define PA metrics or standardized cleaning procedures to enable comparison across research studies. This scoping review examined existing literature on cleaning and pre-processing of actigraphy data. *Approach.* The PubMed (US National Library of Medicine), Scopus (Elsevier), and Web of Science: Core Collection (Clarivate Analytics) databases were searched for original studies published in English from 2017–2024. Using Covidence, two reviewers independently screened each article and collected data. *Results.* A total of 102 studies were included for the final analysis. Our results showed substantial heterogeneity in actigraphy devices, data cleaning and pre-processing methods, with some studies using their own algorithmic approaches to generate PA and sleep variables. While some studies used well-established algorithms like Freedson or Cole–Kripke, a large proportion either developed custom methods or did not report sufficient detail to allow replication. This variability highlights the urgent need for standardized reporting and consensus-based protocols in actigraphy data cleaning and pre-processing to allow replication and comparison of findings across studies. *Significance.* This scoping review is the first to differentiate, in a standardized way, between *cleaning* and *pre-processing* practices in actigraphy research and to quantify reporting practices across multiple device types and data processing strategies. Our findings show a critical gap in standardized reporting and offer actionable guidance for both high- and low-resource research settings.

## 1. Introduction

Over the past few decades, actigraphy has become increasingly relevant for measuring patterns of physical activity (PA) and sleep over the course of hours to weeks (Strath *et al* 2013, Smith *et al* 2018). The rise in popularity is partly due to the non-invasive nature of its sensors and its ability to provide continuous assessments of motion in free-living settings (Aadland and Ylvisåker 2015). Actigraphy devices, also called actimetry sensors, are electromechanical motion-sensor detectors (accelerometers) that are encased in small units the size of a wristwatch and sense movements that can then be converted into PA measures (Sylvia *et al* 2014). Evidence of the shift from traditional methods like polysomnography and self-reported activity logs, and from subjective or lab-based measurement tools, towards continuous, real-world activity tracking using actigraphy is reflected in the rapidly increasing number of studies that employ actigraphy devices and new approaches of analyzing actigraphy data (Migueles *et al* 2017, Smith *et al* 2018).

The use of actigraphy for PA and sleep studies has resulted in the generation of clinically relevant variables. In PA research, the most common variables generated from actigraphy include total steps, activity counts, time spent in different intensity levels (e.g. light, moderate, vigorous), energy expenditure (EE), and sedentary time (Troiano *et al* 2008, Migueles *et al* 2017). The intensity of PA is obtained by using activity count (often per minute) cut-points to differentiate between different intensity levels such as sedentary, light PA, and moderate-to-vigorous PA (MVPA) (Treuth *et al* 2004, Hänggi *et al* 2013, Montoye *et al* 2020). EE, measured in metabolic equivalent of task units, is an indication of how much energy the body uses to perform its functions and can be calculated from actigraphy data using equations developed by several different groups (Crouter *et al* 2006, 2012, Aguilar-Farias *et al* 2019). More than two dozen sleep parameters can be generated from actigraphy data, but only a few are often used in sleep research (Fekedulegn *et al* 2020), such as time in bed (TIB), which is the duration a participant spends lying in bed; sleep onset latency (SOL), which refers to the time it takes a person to fall asleep after lying down in bed; sleep efficiency (SE) which can be calculated in different ways but refers to the percentage of time spent asleep compared to the time spent just lying in bed; total sleep time (TST), which is the total number of minutes spent asleep while lying in bed; and wake after sleep onset (WASO), which is the time a person is awake between the onset and offset of sleep (Marino *et al* 2013, Fekedulegn *et al* 2020). In addition to these variables, the number and duration of awakenings during a sleep period are also commonly used (Berger *et al* 2005, Fekedulegn *et al* 2020).

However, despite the widespread use of actigraphy, data processing practices, particularly in cleaning and pre-processing of actigraphy data, remain highly heterogeneous. Over the years, new approaches have spawned from the traditional idea of ‘activity counts,’ which were often determined from software algorithms provided by the companies that produced popular devices. The rapid proliferation of devices and custom processing methods in both commercial and research settings have introduced considerable variability in how raw actigraphy data are handled. This variability is especially problematic when device-specific processing algorithms are opaque or poorly documented. Despite the attention on actigraphy and abundance of proposed processing techniques, there is still no standardized method of preparing actigraphy data for analysis that is universally accepted or widely adopted.

For this review, cleaning was defined as the removal or correction of raw data artifacts to improve data quality such as excluding non-wear time and reducing the noise of the data. Pre-processing was defined as the transformation of data into a usable format through steps such as re-epoching, generating derived activity metrics, identification of valid sleep windows, and application of scoring algorithms. Techniques to accomplish these tasks vary greatly among studies though. For example, there are several different published algorithms that can be employed to detect non-wear such as the Choi *et al* (2011), Troiano *et al* (2008), or GGIR algorithms (Hees *et al* 2013). Apart from algorithms, researchers have used other techniques such as simply using self-reported logs or removing periods with zero counts (Hutto *et al* 2013, Vanhelst *et al* 2019).

Recent literature has called attention to the lack of consensus on how actigraphy data should be cleaned, filtered, and transformed for analysis (Berger *et al* 2008, Beechy *et al* 2012, Smith *et al* 2018). Standardized procedures for identifying non-wear time, applying filtering algorithms, re-epoching, and setting inclusion thresholds are often either inconsistently reported or not reported at all (Migueles *et al* 2017). These gaps reduce the reproducibility of findings and complicate cross-study comparisons, which is particularly important in large-scale PA and sleep studies. In addition, few reporting frameworks exist to guide reporting clarity for actigraphy-based research (Fekedulegn *et al* 2020).

Recent developments in open-source actigraphy processing pipelines, such as GGIR and ACTman, are helping to improve transparency and reproducibility in the field (Migueles *et al* 2019, Kunkels *et al* 2020, Buchan and Baker 2023). These platforms offer freely available, standardized tools for cleaning, scoring, and analyzing raw accelerometer data, reducing dependence on proprietary software and enabling consistent processes and methods across studies. As these tools become more widely used, they have the potential to support broader efforts to make data processing and reporting more consistent in PA and sleep research.

Therefore, the purpose of this scoping review was to identify and summarize the most commonly reported methods for cleaning and pre-processing raw actigraphy data to generate research variables related to PA and sleep. In doing so, we aim to provide a resource for researchers working in a variety of settings, including those with limited access to proprietary software or computational infrastructure. The findings may also help inform future development of consensus-based reporting guidelines for actigraphy-based studies.

While prior reviews have cataloged actigraphy methods within specific populations or devices (Evenson *et al* 2015, Clevenger *et al* 2019, Haghayegh *et al* 2019, Tazawa *et al* 2019, Fuller *et al* 2020, Huang *et al* 2023, Suau *et al* 2024, Yuan *et al* 2024), this scoping review uniquely measures the reporting of cleaning and pre-processing steps across a wide range of devices, populations, and research designs. By clearly separating and examining these two key steps—*cleaning* (removing errors) and *pre-processing* (organizing the data)—our review provides a structured framework for evaluating how actigraphy data is handled and identifies key areas where reporting still remains inconsistent, unclear, or missing. This broad view is important for advancing transparency, reproducibility, and eventual consensus in actigraphy-based PA and sleep research.

## 2. Methods

### 2.1. Methods, protocol and registration

**Figure 1.**
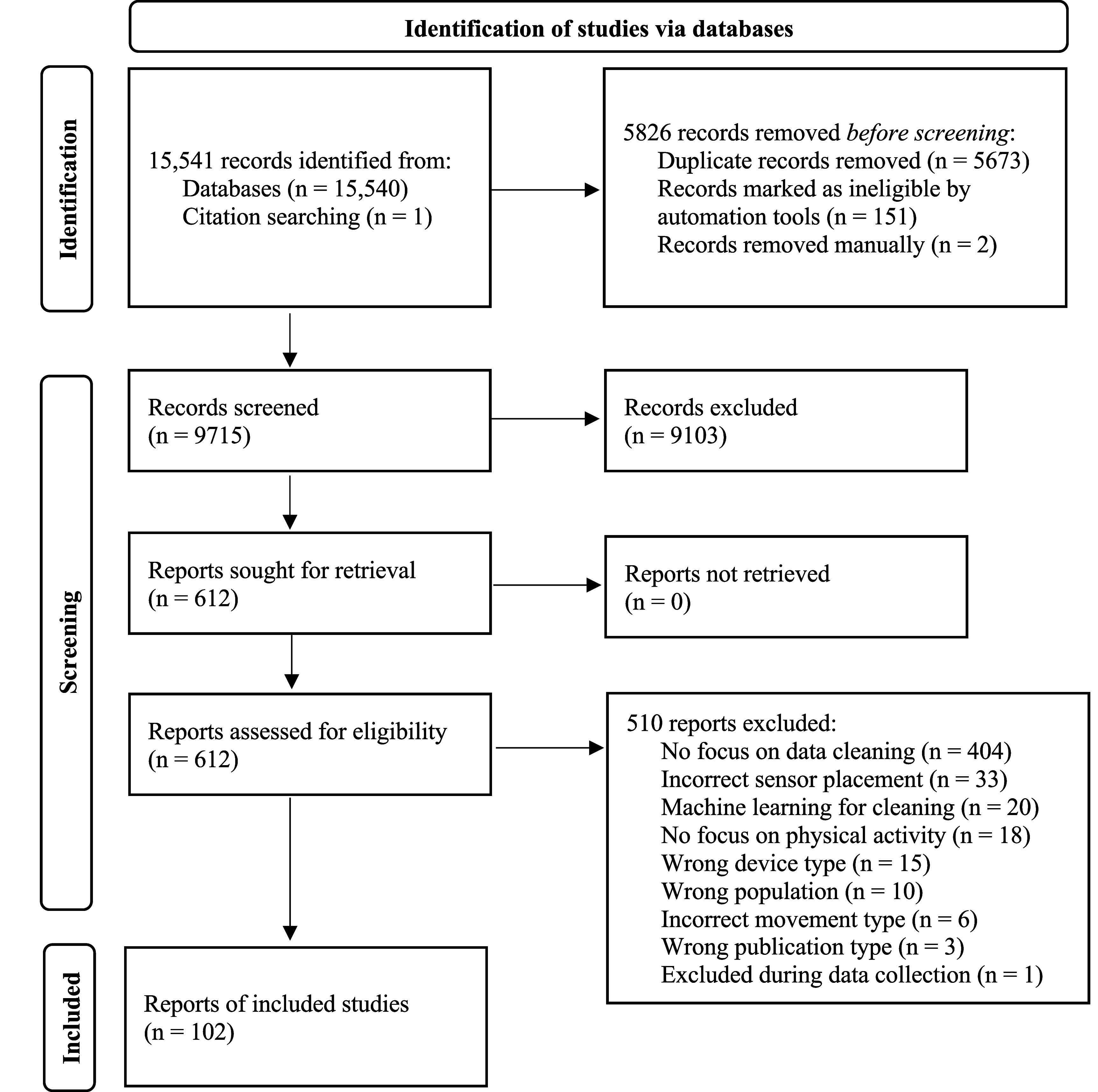
PRISMA flow diagram.

This scoping review was conducted under the guidelines of the Joanna Briggs Institute’s methodology (Peters *et al* 2015), and reported using the Preferred Reporting Items for Systematic reviews and Meta-Analyses extension for Scoping Reviews (PRISMA-ScR, see figure 1) checklist (Tricco *et al* 2018). The protocol was registered on Open Science Framework at the following link: https://doi.org/10.17605/OSF.IO/K8QVP.

### 2.2. Eligibility criteria

Table 1 includes the eligibility criteria used for screening the titles and abstracts of articles retrieved from the database searches.

**Table 1.** Eligibility criteria for title and abstract screening.

Inclusion criteria		Exclusion criteria	
Category	Criteria	Category	Criteria
1. Study population	Human adults (>18 years old). If the population’s age is not mentioned, the article will be included	1. Missing abstracts	Articles without abstracts

2. Movement type	Human whole-body physical activity (i.e., entire body movement such as walking, skipping, jogging, and not just movement of one part or section(s) of the body, as in arms, feet, wrist, head, eyelids, etc.)	2. Study population	If study population <18 years of age, or of mixed ages (e.g. includes both adults, adolescents, and/or pediatrics)

3. Activity type	Article mentions activity monitor or device as actigraphy, tri-axial accelerometer, accelerometry, movement tracker, energy expenditure measuring device, or a synonym of any of these words	3. Study subject(s)	Actigraphy studies of non-human subjects (e.g. wind turbines, robots, weather, earthquakes, continents, navigation, ocean, geologic processes, buildings, bridges, traffic, and inanimate objects)

4. Publication type	Original research, methods and/or validation papers, pilot studies, and frameworks. If publication type is not clear, the paper will be included.	4. Device types	Articles that mention using actigraphy for non-movement specific variables (e.g. body temperature, pulse ox, tremors), and/or to augment movement collection—besides whole body—such as muscle activity by EMG, heart rhythm by EKG, brain activity by EEG, eye movement by EOG, geographical location tracking (GPS), and 3D models of human movement

5. Language	English only	5. Sensor types	Movement ‘sensors’ other than accelerometers/actigraphy such as machine vision-based cameras, floor/step sensors, optical gait analysis sensors, pedometers, or step counters

6. Data processing/analysis	Methodology and/or results section should mention data cleaning of human physical activity data, data scrubbing, data quality control, preparing data sets, data cleansing, and data pruning, or a synonym thereof. If data cleaning is not mentioned, the article will be included. a. Data must be related to variables specific to movement; and b. Data cleaning must be on raw/unmodified data	6. Publication types	Reviews (narrative, systematic, and meta-analysis), editorials, letters, commentary, books and/or book chapters, dissertations, conference abstracts and proceedings, case studies, case reports, opinion papers, posters, and briefs as well as grey literature and information produced outside of traditional publishing and distribution channels (e.g., reports, policy working/white papers, newsletters, speeches), and articles that are not empirical (i.e., only from theory) or that did not provide enough information for our analysis

7. Publication date	2017–2024	7. Study type	Animal and *in vitro* studies, as well as articles in which results were exclusively qualitative.

For full text screening, additional eligibility criteria were added to further specify the device location or type, activity type, and sleep or inactivity to be included (table 2).

**Table 2.** Eligibility criteria for the full text screening.

Inclusion criteria		Exclusion criteria	
Category	Criteria	Category	Criteria
1. Device Type/location	Only studies which use an accelerometer (not pedometers or step counters) on the wrist and waist areas will be included	1. Primary article subject and variables	Articles were excluded if they analyzed actigraphy data without reporting at least one raw-data cleaning or pre-processing step (e.g. non-wear detection, artifact removal, epoching, or signal filtering). Manuscripts reporting only derived PA/sleep outcomes (including sedentary time) with no cleaning/pre-processing description were excluded.

2. Activity type	Human physical activity data from any type of whole bodily movement	2. Movement type	Full text articles that do not include human, whole-body movement data. Studies specific to posture, gait, balance, tremors, gestures, falls, and fall detection systems (these activity patterns require algorithms, thresholds, filters, and statistical analysis different from typical physical activity) and special populations where movement is altered or impaired (e.g. lower limb amputees, wheelchair bound, casted foot or ankle fractures, cerebral palsy, etc)

3. Inactivity/sleep	Studies that measure activity frequency, duration, and intensity to determine sedentary behaviors (SBs), and sleep-wake periods—in addition to PA—and distinguish times spent in each will be included	3. Activity type	Articles that do not define wear versus non-wear and active versus sedentary activity

		4. Sensor placement	Studies which only use activity monitors/accelerometers in areas besides hip and wrist (e.g., 3D chest-mounted accelerometer, foot, ankle, back, head, etc.)

		5. Device type	Actigraphy/accelerometers from cell phones and pedometers.

		6. Publication type	Studies that use algorithms as combinations of accelerometers and gyroscopes attached to different body locations; unless specific procedure(s) are used to clean and score raw accelerometer data separately, and devices that pair accelerometers with other devices to measure non-motor activities (e.g. Brain activity EEG, Eye EOG, muscle EMG, heart ECG).

		7. Study type	Modeling, framework, and methodology studies (all without primary outcome of data cleaning) articles in which results were exclusively qualitative, or where no PA data was collected and/or analyzed

		8. Algorithms	Articles that do not describe the activity detection algorithms and/or data processing (cleaning) and analysis (scoring) methods, or if it is a proprietary algorithm, the method(s) used to clean and score raw data, or if no preprocessing of PA data was done, and AI or machine learning (i.e., neural networks, random forest (RF) classifier, human activity recognition) systems that preprocess the data.

### 2.3. Information sources and search strategy

A biomedical librarian (AAL) searched three online databases: PubMed (US National Library of Medicine), Scopus (Elsevier), and Web of Science: Core Collection (Clarivate Analytics) in April 2022 and with updates in June and August 2024. Additionally, the bibliographies of the records included after full-text screening and relevant reviews were reviewed by the team members and potentially relevant citations collected. The librarian then compared these collected records against the database search results and the unique records were then added for screening using the process described below.

The librarian developed the search strategy with feedback from the review team. A combination of keywords and controlled vocabulary terms (i.e., MeSH for PubMed) were used to describe the concepts of interest (i.e., actigraphy and data cleaning). The final search strategies used are in supplemental file 1. The searches were limited to articles published between 2017 and 2024 to capture more recently used devices, written in the English language, published as original research articles (excluded editorials, letters, case studies, case reports, conference abstracts, conference proceedings, commentary, protocols, reviews, errata, and corrigenda), and utilized human subjects.

### 2.4. Study selection

Covidence (Veritas Health Innovation Ltd, Melbourne, Australia) was used for study screening. Study selection occurred in two rounds with two independent reviewers screening each record. The first round involved the screening of titles and abstracts based on the eligibility criteria in table 1. The second round involved the full text screening of those articles included from round 1. Additional eligibility criteria focused on the relevance of the study to our research questions were used for round 2 (table 2). Any conflicts among reviewers were discussed with the entire review team and a consensus decision was applied.

Prior to commencing the review, two rounds of piloting the screening steps were conducted on a total of 77 records randomly selected and added to Covidence by the biomedical librarian. All reviewers participated in the screening pilot. After the pilot, all conflicts were discussed and any revisions or clarifications to the eligibility criteria were made and documented in the protocol.

### 2.5. Data collection and data items

Two independent reviewers collected data from each included article based on a pre-defined collection form in Covidence (supplemental file 2). An expert third reviewer (KC) resolved any discrepancies in the collected data between the two reviewers and ensured that other questions were answered accurately before approving the extracted data.

A pilot of the data collection process was completed with all reviewers using the 8 articles included after the screening pilot. Discrepancies were discussed and resolved by the entire review team and as needed, the data collection form in Covidence was updated.

The data collected from each included article was general citation information, study population size and type, study design, type of PA studied, criteria used to categorize PA, actigraphy data collection criteria (e.g., placement, sampling frequency, epoch length, non-wear time), actigraphy cleaning method used, actigraphy algorithms used, actigraphy variables reported, non-wear/sedentary times descriptions, and other general comments. See supplemental file 2 for more details.

### 2.6. Risk of bias

The risk of bias for all included studies was not examined as the research questions were focused on data cleaning and pre-processing methodology rather than the conclusions that arise from the studies themselves.

### 2.7. Data synthesis

A narrative summary with tables and figures will be presented including the PRISMA flow diagram. Answers to the data extraction form were grouped into larger categories if a common theme was found.

## 3. Results

The literature searches resulted in 15 541 records of which 5826 were removed (i.e., duplicates, records not meeting criteria) and 9715 unique records underwent screening. Of these, 9103 studies were excluded and 612 proceeded to full text screening where 509 studies were further excluded. A total of 103 records underwent data collection and one record was excluded because of irrelevance to PA or sleep, resulting in 102 included articles for the final analysis.

### 3.1. Study selection and characteristics

The years of publication spanned eight years, from 2017 to 2024, with the highest number of studies (21%, *n* = 21) published in 2020. Most studies included participants of both sexes (80.4%, *n* = 82). A smaller proportion focused exclusively on females (*n* = 12, 11.8%), while two studies enrolled male participants only (2.0%). Six studies (5.9%) did not report the sex of participants. Participants in the included studies varied in health conditions: 38 studies enrolled healthy population, 20 were disease-specific, 21 used a mix of healthy and disease-specific individuals, and 23 did not report the health status of their participants.

The average sample size was 3071 participants (range = 3–96 600 from *n* = 101 studies), and one study did not report its population size. The sample sizes varied widely, from small-scale studies to large cohorts: the median sample size was 78, with an interquartile range of 41–289. When excluding studies with sample sizes greater than 10 000 (*n* = 96), the median was 71, and the IQR narrowed to 39–181. Several studies included large-scale population datasets such as National Health and Nutrition Examination Survey (NHANES) and the UK Biobank, which contributed to the wide range and high variability in sample sizes observed across the included studies.

Most studies were for validation and method development (*n* = 57) followed by cross-sectional (*n* = 42); very few were longitudinal observational (*n* = 5), longitudinal interventional (*n* = 3), descriptive (*n* = 5), and experimental (*n* = 1). In terms of where studies were conducted, the vast majority assessed activity counts in free-living settings (*n* = 80), which meant participants wore the actigraphy devices as they went about their normal life. Some studies were conducted in the laboratory (*n* = 15), and few were conducted in a participant-chosen location (*n* = 1), researcher-chosen location (*n* = 4), clinic (*n* = 4), other locations not specified above (*n* = 2), or multiple settings (*n* = 8), while 4 studies did not report their study setting.

### 3.2. Actigraphy device characteristics: models, placement, and sampling rates

A variety of accelerometers were used (see table 3); in fact, some studies (*n* = 20) employed multiple devices simultaneously (e.g., wrist + hip) to assess study outcomes. ActiGraph^7^7ActiGraph LLC, Pensacola, Florida, USA. was the most commonly used device (*n* = 54), followed by ActiWatch^8^8Philips Respironics, Murrysville, Pennsylvania, USA. (*n* = 13), GENEActiv^9^9ActivInsights Ltd, Kimbolton, Cambridgeshire, UK. (*n* = 11), Fitbit^10^10Google Fitbit, San Francisco, California, USA. (*n* = 10), and Axivity^11^11Axivity Ltd, Newcastle Helix, Newcastle upon Tyne, UK. (*n* = 5). A number of studies (*n* = 28) used other unique devices not included in these categories (e.g., Garmin Vivosmart or Empatica E4 wristband)^12^12e.g. Garmin Vivosmart (Garmin Ltd, Olathe, Kansas, USA), Empatica E4 (Empatica Inc, Boston, Massachusetts, USA)., and two studies did not report the device type.

**Table 3.** Accelerometer devices used in included studies.

Device Name	Manufacturer/notes	Number of studies
ActiGraph	ActiGraph LLC (USA)^1^	54 (52.9%)
Other/unique devices	e.g. Garmin Vivosmart, Empatica E4^6^	28 (27.5%)
Used multiple devices	Used > 2 devices (e.g., wrist + hip)	20 (19.6%)
ActiWatch	Philips Respironics (USA)^2^	13 (12.7%)
GENEActiv	ActivInsights Ltd (UK)^3^	11 (10.8%)
Fitbit	Google Fitbit (USA)^4^	10 (9.8%)
Axivity	Axivity Ltd (UK)^5^	5 (4.9%)
Not reported	Device not specified	2 (2%)

*Note*: Total exceeds 102 because some studies used more than one device.

The primary placement of actigraphy devices was either on the non-dominant wrist (*n* = 45) or right hip (*n* = 23). Placement affects the sensitivity and interpretation of movement data, making it a key consideration in PA and sleep study design (Quante *et al* 2015, Wennman *et al* 2019, Bate *et al* 2023). Apart from the wrist or hip, some studies also used secondary placements (*n* = 20). Wrist and hip placements are favored because they balance participant comfort with reliable data collection, the wrist for continuous wear and sleep monitoring, and the hip for accurate estimation of ambulatory PA (Ellis *et al* 2014, Zinkhan *et al* 2014, Gall *et al* 2022).

The most common sampling rates were 30 Hz (*n* = 25), 100 Hz (*n* = 18), and 32 Hz (*n* = 11), with 14 studies not reporting on their sampling rates. The remaining studies used various other sampling rates such as 80 Hz (*n* = 6), 60 Hz (*n* = 3), 64 Hz (*n* = 3), 50 Hz (*n* = 3), 1 Hz (*n* = 2), 200 Hz (*n* = 1), 25 Hz (*n* = 1), and 20 Hz (*n* = 1). Eleven of the studies also used multiple sampling rates. Sampling rates are an important consideration in actigraphy studies because higher frequencies (e.g., 100 Hz) provide higher sampling resolution and more detailed motion capture, which can improve the accuracy of the processed variables (Lettink *et al* 2024). However, higher sampling rates also increase data volume, which can place greater demands on device storage, battery life, and post-processing resources (Sasaki *et al* 2016). For most free-living adult studies, sampling frequencies of 30–100 Hz balance temporal resolution with battery life. Higher frequencies (⩾80 Hz) are advisable for short-epoch or gait analyses, whereas lower frequencies (<30 Hz) may miss micro-movements and are not recommended for high-intensity or sleep-fragmentation research.

### 3.3. Cleaning and pre-processing

Cleaning refers to the removal or correction of raw data artifacts to ensure data integrity before further analysis (Broeck *et al* 2005). The techniques used by the reviewed studies included removal of non-wear time (*n* = 62), exclusion of invalid time periods (e.g. device removal or artifact-prone intervals, *n* = 21), calibration or auto-calibration (*n* = 9), visual inspection (*n* = 10), threshold-based exclusions (*n* = 8), and consistency checks (*n* = 1). Previous studies have highlighted a range of cleaning techniques commonly used in actigraphy research, such as removal of non-wear time, exclusion of artifact-prone periods, and application of threshold-based rules (Migueles *et al* 2017). In our review, we found that while some of these techniques—like visual inspection (*n* = 10), calibration (*n* = 9), and threshold-based exclusions (*n* = 8)—were used across studies, others such as consistency checks (*n* = 1) were rare or under reported. Additionally, removal of specific time periods (*n* = 21), often related to known non-wear or artifacts, was one of the most common cleaning steps in the reviewed studies. A substantial number of studies employed custom or study-specific approaches (*n* = 31), such as interpolation or unclassified filters, while 25 studies did not report their cleaning procedures. This lack of clear reporting shows a widespread gap in how cleaning methods are described across studies.

Pre-processing involves transforming the cleaned data into formats suitable for analysis, such as re-epoching, data aggregation, and feature extraction (Gabriel *et al* 2010, Fuster-Garcia *et al* 2015, Ortiz *et al* 2024). Signal filtering methods, often referred to as ‘cleaning’ in some literature, are technically pre-processing steps that shape the data for further analysis (Hees *et al* 2013). As listed in table 4, the filtering approaches used by the reviewed studies included Butterworth filters (n = 37), low-frequency extension (LFE) filters (*n* = 11), low-pass filters (*n* = 10), band-pass filters (*n* = 8), and high-pass filters (*n* = 7). These filters reduce noise or isolate specific frequency bands in the acceleration signal to help interpretation.

**Table 4.** Initial cleaning techniques used in included studies.

Cleaning method	Category	Number of studies
Butterworth filters	Signal filter	37 (36.3%)
Removal of specific time periods	Manual/data-driven	21 (20.6%)
Low frequency extension (LFE) filters	Signal filter	11 (10.8%)
Low pass filters	Signal filter	10 (9.8%)
Visual inspection	Manual/data-driven	10 (9.8%)
Calibration/auto-calibration	Manual/data-driven	9 (8.8%)
Removal of data above/below thresholds	Manual/data-driven	8 (7.8%)
Band pass filters	Signal filter	8 (7.8%)
High pass filters	Signal filter	7 (6.9%)
Consistency checks	Manual/data-driven	1 (1%)
Custom/specialized cleaning (e.g., interpolation)	Study-specific	31 (30.4%)
Not Reported	Method not specified	25 (24.5%)

*Note:* 102 studies reported 153 cleaning methods, indicating at least 51 studies used more than one initial cleaning technique.

### 3.4. Epoch length and aggregation

Following cleaning and filtering, accelerometer data were commonly re-epoched into standardized time intervals to facilitate comparison across studies and participants. This is a core pre-processing step aimed at summarizing continuous acceleration data into consistent, time-binned measures (Migueles *et al* 2017). Of the included studies, 22 used short epochs between (1–15 s), five used moderate epochs (30–60 s), 56 articles used either 60 s or daily epochs, 11 used combinations of short and long epochs, and eight studies did not report their epoch length. Shorter epochs allow for finer resolution of activity patterns and may better detect brief bouts of movement compared to longer intervals (Dorsey *et al* 2009, Ayabe *et al* 2013).

### 3.5. Non-wear detection methods

Accurately identifying and characterizing non-wear time is essential for ensuring the valid interpretation of actigraphy data in PA and sleep studies (Choi *et al* 2011). Misclassified non-wear periods—such as when a participant removes the device—can be mistaken for sleep or sedentary behavior, which can lead to overestimating inactivity or underestimating PA and sleep time (Zhou *et al* 2015, Draper *et al* 2020, Vert *et al* 2022). This is especially critical in free-living studies, where the participants’ compliance and behavior are not directly observed (Vert *et al* 2022). Applying transparent and consistent non-wear detection methods improves data quality, reduces misinterpretation, and enhances reproducibility across studies (Knaier *et al* 2019). It also helps set clear rules for wear-time, which makes results more consistent and ensures that the data accurately reflects real behavior or PA (Draper *et al* 2020).

Approaches to identifying non-wear time varied considerably across studies (see table 5). Many researchers (*n* = 38) did not report the method used. Among those that did, the Choi algorithm was the most applied (*n* = 23); it determines non-wear based on three criteria: (1) non-zero count thresholds, (2) time windows for consecutive zero/non-zero counts, and (3) the presence of artifactual movements (Choi *et al* 2011). Other commonly used methods included self-reported activity logs (*n* = 17), and zero-count detection (*n* = 15), which classifies sustained low or no movement as non-wear (Choi *et al* 2011, Hutto *et al* 2013, Vanhelst *et al* 2019). Fifteen studies applied alternative or study-specific methods, such as the DETACH algorithm, which uses temperature and motion signatures to detect device removal (Vert *et al* 2022).

**Table 5.** Non-wear algorithm usage summary.

Non-Wear Algorithm	Number of studies
Choi	23 (22.5%)
Self-report (logs)	17 (16.7%)
Zero counts	15 (14.7%)
Other techniques (e.g., DETACH)	15 (14.7%)
Troiano	10 (9.8%)
Temperature/light sensors	6 (5.9%)
GGIR *z*-angle	5 (4.9%)
Manual/visual inspection	4 (3.9%)
Threshold-based methods	1 (1%)
Not Reported	38 (37.3%)

*Note*: The total number of included studies is 102. Many studies used more than one non-wear detection algorithm, so counts and percentages may sum to more than 100%. Of the 102 included studies, 38 did not report a non-wear detection method. Among the 64 that did, many used more than one approach, resulting in 135 total methods. This indicates that the use of multiple non-wear algorithms within a single study was common.

Additional non-wear detection methods included the Troiano algorithm (*n* = 10), which identifies non-wear as 60 consecutive minutes of zero counts, allowing brief interruptions of minimal activity (Troiano *et al* 2008), and sensor-based detection (*n* = 6), using temperature or light signals to infer removal. The GGIR *z*-angle algorithm (*n* = 5) uses variation in arm angle to detect non-wear (Hees *et al* 2011, 2013). Less frequently reported methods included manual inspection (*n* = 4) and threshold-based rules (*n* = 1).

*Comparative strengths and limitations of non-wear algorithms*: The Choi algorithm offers validated count-based detection but may miss brief removals. Troiano is ideal for large-scale datasets but less sensitive to short interruptions. GGIR *z*-angle integrates raw acceleration (and, when available, additional signals such as temperature), enhancing accuracy for wrist-based wear. Sensor- or light-based detection can improve precision but requires multi-channel devices. Manual inspection remains a useful reference but is impractical for high-volume data.

### 3.6. Daily and study-level wear-time criteria

Once non-wear time was identified using these methods, studies applied specific thresholds to determine whether a day or participant met criteria for inclusion in the analysis. The amount of wear time required to include a day or participant in analysis has important implications for data quality. If thresholds are too strict, valid data may be excluded; if too lenient, periods of non-wear or inactivity may be misclassified as valid, reducing accuracy (Knaier *et al* 2019).

After identifying non-wear time, 39 studies applied fixed-hour requirements to define a valid day (e.g., requiring a minimum number of hours worn per day). A few studies used alternative daily criteria, including percentage of the day worn (*n* = 3), hourly distribution across the day (*n* = 2), sleep-specific requirements (*n* = 3), or exclusion-based rules (*n* = 4). However, more than half of the studies (*n* = 52) did not report any daily wear-time threshold. For study-level inclusion, 50 studies also did not report the minimum number of valid days required. Among those that did, 46 studies used a fixed number of days as an inclusion threshold, while others required weekday and weekend representation (*n* = 4), a certain number of nighttime recordings (*n* = 1), or used additional exclusion criteria (*n* = 1). In summary, wear-time criteria varied widely across studies, and many failed to report them altogether, limiting comparability and replication across actigraphy-based research.

### 3.7. Activity metrics derived from acceleration data

Reviewed actigraphy studies used a range of metrics derived from accelerometer data, broadly falling into two categories: device-specific processed outputs and raw acceleration-derived metrics (see table 6). Activity counts, which are the most commonly used metric, were reported in 39 of the 102 reviewed studies. They refer to device-specific units calculated over time intervals using proprietary algorithms provided by the manufacturer. These counts quantify acceleration within a fixed epoch and may differ in calculation between devices. Another 28 studies reported using general activity counts without specifying the axis measured. In contrast, metrics derived from raw acceleration data, which are typically device-independent and reproducible across platforms, included Euclidean Norm Minus One (ENMO, *n* = 14), the mean of raw acceleration (*n* = 14), and the standard deviation or variance of raw acceleration (*n* = 12). Less frequently used metrics in this category included signal vector magnitude (SVM, *n* = 8), minimum/maximum acceleration (*n* = 7), percentiles of raw acceleration (*n* = 5), mean amplitude deviation (MAD, *n* = 4), and dominant frequency (*n* = 4). Four studies did not report the specific metrics used. Additionally, 39 studies used other unique or study-specific metrics. For example, one study used a modified ENMO formula, another used a modified activity count formula, and a third study applied a directional change metric.

**Table 6.** Activity metrics used in included studies.

Metric type	Number of studies
Activity counts (proprietary)	39 (38.2%)
General activity counts (axis not specified)	28 (27.5%)
Euclidean norm-minus one (ENMO)	14 (13.7%)
Mean of raw acceleration	14 (13.7%)
Standard deviation/variance of raw acceleration	12 (11.8%)
Signal vector magnitude (SVM)	8 (7.8%)
Min/max of raw acceleration	7 (6.9%)
Percentiles of raw acceleration	5 (4.9%)
Mean amplitude deviation (MAD)	4 (3.9%)
Dominant frequency of raw acceleration	4 (3.9%)
Not reported	4 (3.9%)
Other/study-specific metrics (e.g., directional change metrics)	39 (38.2%)

*Note:* Most studies reported more than one metric. *Interpretive comparison of activity metrics:* proprietary activity counts enable continuity with legacy datasets but hinder cross-device reproducibility. Raw-acceleration metrics such as ENMO and MAD are transparent and device-independent, supporting harmonized analyses, but they require access to raw tri-axial data and additional computational preprocessing. Adoption of open, raw-based metrics may improve future meta-analytic synthesis.

### 3.8. PA outcomes and processing

The methods used to generate PA variables from actigraphy data varied greatly. In fact, many studies (*n* = 45) used techniques that appeared only once or twice across the 102 reviewed articles. For example, some used Montoye’s 2020 cut-points (*n* = 1, sedentary <2860 counts/min, light 2860–3940 counts/min, and MVPA ⩾3941 counts/min) (Montoye *et al* 2020) or developed custom cut-points specific to their population (*n* = 3). The most-applied standardized methods to process PA outcomes were Freedson cut-points or equations (*n* = 19) (Freedson *et al* 1998), followed by proprietary algorithms (*n* = 14), and other machine learning techniques (*n* = 16). Other named standards included Sasaki cut-points (*n* = 9) (Sasaki *et al* 2011), Troiano (*n* = 5) (Troiano *et al* 2008), Kozey-Keadle (*n* = 4) Kozey-Keadle *et al* 2011), and Matthews (*n* = 3) (Matthew 2005), while only one study did not report its method.

The use of such diverse and often study-specific cut-points limits direct comparability across studies, particularly when similar intensity labels (e.g., ‘moderate activity’) may correspond to widely different count thresholds. Only 27% of studies (*n* = 28) used commonly accepted and well-documented thresholds such as Freedson, Sasaki, or Troiano, which further challenges comparisons. These differences mean that the same raw acceleration data can yield different interpretations of PA intensity depending on the cut-point system applied. This directly influences PA summary variables such as minutes per day in MVPA or time spent sedentary and ultimately impacts comparisons across studies. Although some studies applied machine learning approaches to classify PA intensities (*n* = 6), these methods were not validated or compared against established cut-point systems in the studies we reviewed. As our review focused on identifying common cleaning and pre-processing approaches used to generate actigraphy-derived PA and sleep variables, the validation of the classification methods was outside the scope of our analysis. Table 9a summarizes cleaning and pre-processing practices used in PA research, including device type, non-wear detection approaches, daily/study-level wear criteria, and cut-point selection.

### 3.9. Sleep outcomes and scoring algorithms

Most articles (*n* = 71) did not report any sleep-related outcomes. As listed in table 7, among the 31 studies that did, the most reported variables were TST (*n* = 26), SE (*n* = 16), WASO (*n* = 15), and SOL (*n* = 12). Less frequently reported were number of awakenings (*n* = 6) and TIB (*n* = 5). The remaining studies (*n* = 18) generated other sleep-related variables that appeared too infrequently to categorize. For example, a few studies reported measures such as interdaily stability (*n* = 2), intradaily variability (*n* = 2), or average awakening length (*n* = 2), which may reflect attempts to capture more complex aspects of sleep-wake patterns.

**Table 7.** Sleep outcomes generated from reviewed studies.

Sleep outcome	Number of studies	Sleep outcome processing	Number of studies
Total sleep time (TST)	26 (25.5%)	ActiWatch or Actical thresholds	9 (8.8%)
Sleep efficiency (SE)	16 (15.7%)	Cole–Kripke	8 (7.8%)
Wake after sleep onset (WASO)	15 (14.7%)	GGIR	6 (5.9%)
Sleep onset latency (SOL)	12 (11.8%)	Sadeh	3 (2.9%)
Number of awakenings	6 (5.9%)	Tudor-Locke	1 (1%)
Time in bed (TIB)	5 (4.9%)	Proprietary algorithms	2 (2%)
Other outcomes	18 (17.6%)	Other algorithms	18 (17.6%)
Not reported	71 (69.6%)	Not reported	71 (69.6%)

*Note*: Most studies used more than one sleep outcome.

A total of 18 studies applied algorithms or computational techniques to generate sleep-related variables. These included custom algorithms or deep learning methods developed specifically for the study. Table 9b summarizes methods in sleep-focused actigraphy research, including device placement, wear-time criteria, cleaning approaches, and sleep outcome algorithms. Among the standardized tools, the most used were ActiWatch or Actical sleep-wake thresholds (*n* = 9), followed by the Cole–Kripke algorithm (*n* = 8), which is widely used for sleep-wake classification and validated in adult populations (Cole *et al* 1992). Other methods included the GGIR algorithm (*n* = 6), which estimates sleep using the variance in the *z*-axis angle derived from raw acceleration data (Van Hees *et al* 2018), and the Sadeh algorithm (*n* = 3), originally developed for children but later validated for adolescents and adults (Sadeh *et al* 1994). Additionally, proprietary algorithms provided by commercial vendors were used in two studies, while one study applied the Tudor-Locke algorithm, which identifies ‘bedtime’ and ‘wake time’ and can integrate multiple sleep episodes across a 24 hour period (Tudor-Locke *et al* 2014).

## 4. Discussion

Across the 102 included studies, there was substantial variability in devices, data cleaning, pre-processing, and algorithmic approaches used to generate PA and sleep variables (see table 6). Although actigraphy remains a widely adopted tool for non-invasive monitoring of free-living behaviors, the field is challenged by a lack of consensus on standard protocols and inconsistent reporting practices. These inconsistencies undermine the comparability, reproducibility, and interpretability of actigraphy-derived findings across studies, as has been previously noted in the literature (Morgenthaler *et al* 2007, Berger *et al* 2008, Beechy *et al* 2012, Smith 2015, Smith *et al* 2018). Given the central role these thresholds play in ensuring data validity, their omission significantly hinders reproducibility. This variability highlights the urgent need for standardized reporting and consensus-based protocols in actigraphy data cleaning and pre-processing.

**Figure 2.**
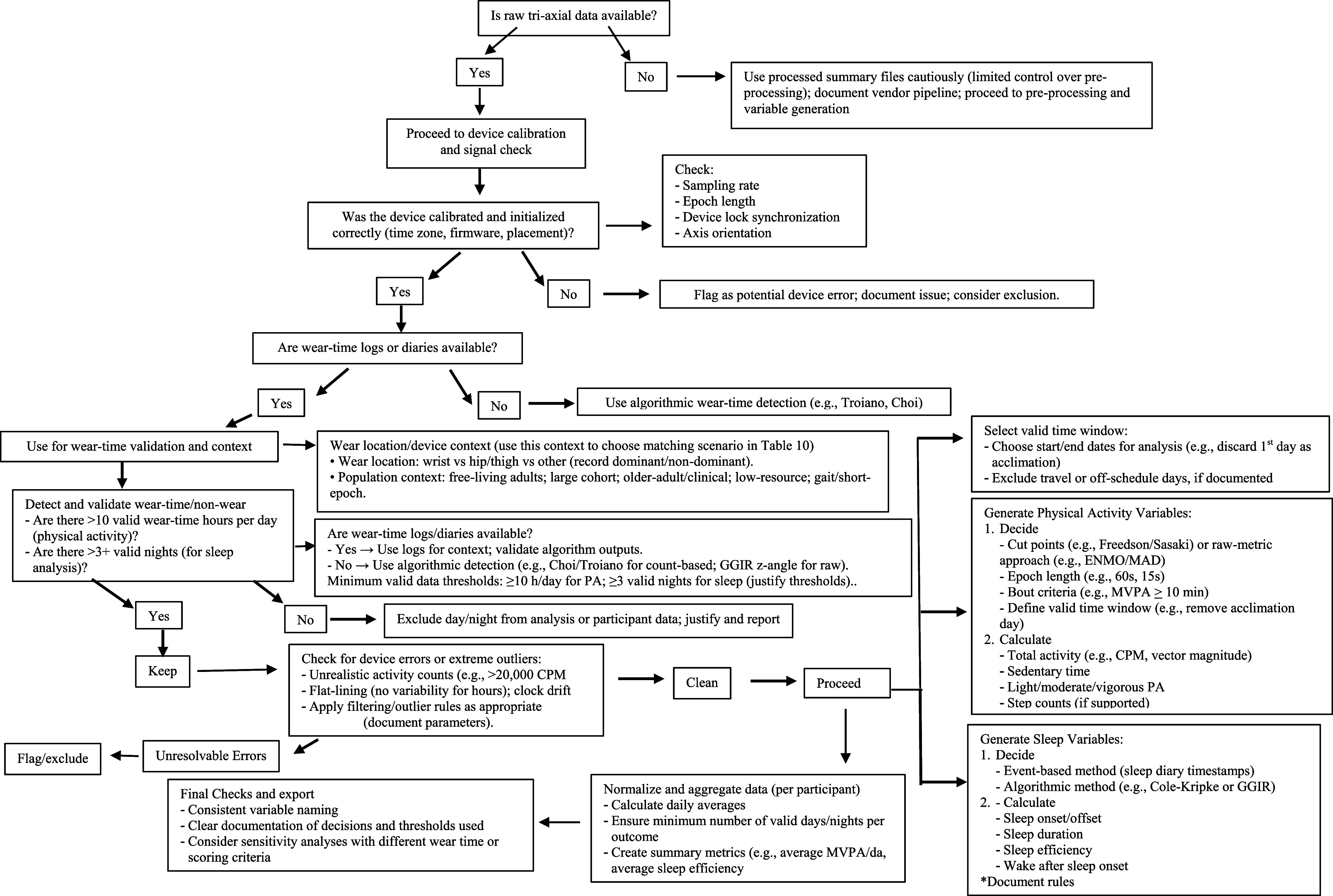
Proposed decision tree for cleaning and pre-processing of actigraphy data for physical activity and sleep research. The tree incorporates conditional branches for (a) device type and wear location, (b) data availability (raw vs proprietary), and (c) study population. Each terminal node references recommended algorithms and corresponding rows in table 10, providing step-by-step guidance from data acquisition to derived metrics.

A prior systematic review by Migueles *et al* (2017) classified studies using the ActiGraph GT3X/+ to assess sedentary time, PA, EE, and sleep, and evaluated data collection and processing methods by age group (Migueles *et al* 2017). While that work offered valuable practical considerations, it was limited to studies published between 2010 and 2015 and focused exclusively on one type of device. Similarly, Gorman *et al* (2014) reviewed accelerometry studies in older adults to examine variation in cut-points for MVPA and sedentary behavior, demonstrating how different processing thresholds can yield markedly different activity estimates (Gorman *et al* 2014). However, their review was restricted to waist-worn ActiGraph devices and did not explore cleaning, non-wear detection, or sleep processing methods. In contrast, our scoping review captured a broader swath of the actigraphy literature from 2017 to 2024, including multiple device types (e.g. ActiGraph, GENEActiv, Fitbit) and analytical approaches. Our review builds on and extends these earlier findings by identifying substantial underreporting of important processing steps such as non-wear detection methods (unreported in 37% of studies), daily wear-time criteria (51% not reported), and cleaning procedures (24% not reported). To provide scenario-driven guidance, we developed a decision tree (figure 2) illustrating conditional workflows for actigraphy data. The tree links study design choices to recommended cleaning and pre-processing decisions, beginning with device calibration and raw data availability and progressing through non-wear detection, variable generation, and final data export.

We also summarize actigraphy processing practices across a broader range of study designs, populations, and device settings. Importantly, we highlight low-resource-compatible methods that can be implemented without proprietary software, making our findings particularly relevant for settings with limited resources. Recognizing the critical need for transparent reporting, we also created a reproducibility checklist (supplementary file 1) that outlines essential reporting elements for actigraphy studies. The checklist covers device specifications, data acquisition parameters, cleaning methods, wear-time criteria, handling of missing data, sleep/wake identification, and software/code availability. This structured tool complements existing reporting guidelines and supports reproducibility across diverse study designs.

Another major finding was the inconsistent application of non-wear detection and wear-time thresholds. Over half of the reviewed studies did not report daily wear-time criteria, and nearly half omitted study-level wear-time inclusion criteria altogether. The diversity in PA cut-points further complicates the synthesis of findings across studies. While Freedson cut-points were the most frequently used, fewer than one-third of studies applied established methods such as Freedson, Sasaki, or Troiano. Many opted for proprietary or machine learning-derived approaches that lacked clear documentation or validation. These inconsistencies can yield conflicting interpretations of activity intensity, even when analyzing the same raw data. It is also important to note that many widely used cut-points were originally developed in younger, generally healthy adults. For studies in older adults or those with chronic illness, these thresholds may not adequately capture true activity intensity, and alternative or population-specific methods may be more appropriate.

Unclear or missing reporting was common across studies. For example, 37% of studies did not mention a non-wear detection method, 51% left out daily wear-time rules, and nearly one-quarter did not explain any cleaning steps. These gaps directly make it harder to repeat the research and combine findings across studies. To enhance future reporting, table 8 shows the most commonly missing details. These missing details show that clear reporting guidelines that match existing efforts to make studies easier to repeat and help combine data across research projects is needed.

**Table 8.** Most common reporting omissions in reviewed studies (*N* = 102).

Reporting element	Not reported	% of studies
Non-wear detection method	38	37.3%
Daily wear-time threshold	52	51.0%
Study-level inclusion criteria	50	49.0%
Cleaning technique	25	24.5%
Sampling rate	14	13.7%
Epoch length	8	7.8%
Device placement	4	3.9%

**Table 9a.** Cleaning and pre-processing techniques of actigraphy data for physical activity research.

Study	Accelerometer device	Initial cleaning technique	Non-wear detection algorithm	Daily wear time criteria	Study level wear time inclusion criteria	Activity metrics	Physical activity cut-points
ActiGraph							

Beale *et al* (2020)	ActiGraph	Removal of data from a specific time	Zero counts	Fixed hour requirements	Fixed minimum days	Activity counts	Custom cut-points; Troiano
Bellettiere *et al* (2019)	ActiGraph	LFE filter	Choi; user-reported data; other	Not reported	Fixed minimum days	Activity counts	Other (Evenson)
Bevier *et al* (2020)	ActiGraph; Fitbit	Not reported	Troiano	Fixed hour requirements	Fixed minimum days	Activity counts	Freedson; proprietray
Brønd *et al* (2017)	ActiGraph; Axivity	High pass filter; low pass filter; band pass filter; other	Not reported	Not reported	Fixed minimum days	Activity counts	Freedson
Christofoletti *et al* (2021)	ActiGraph	Not reported	Choi	Fixed hour requirements	Fixed minimum days	Counts (axis not specified)	Freedson
Chu *et al* (2021)	ActiGraph	Visual inspection	Choi	Fixed hour requirements	Fixed minimum days	Counts (axis not specified)	Kozey-Keadle; Sasaki; Other
Chudyk *et al* (2017)	ActiGraph	Removal of data from a specific time	Choi; Troiano; Zero counts; User-reported data	Fixed hour requirements	Fixed minimum days	Activity counts	Freedson; Matthews
Cook (2019)	ActiGraph	Removal of data from a specific time	Zero counts; other	Fixed hour requirements	Fixed minimum days	Activity counts	Custom cut-points; Other
Ehrlich *et al* (2020)	ActiGraph	LFE filter; Removal of data from a specific time	Choi; Troiano; temperature, light, or other sensors; user-reported data	Fixed hour requirements	Not reported	Activity counts; ENMO	Other (Hibbing *et al* 2018)
Farrahi *et al* (2024)	ActiGraph	Removal of data above/below threshold; visual inspection	Other	Fixed hour requirements	Fixed minimum days	Activity counts	Freedson; Matthews
Gafoor *et al* (2024)	ActiGraph	Not reported	Not reported	Not reported	Not reported	Activity counts	Freedson
Graves *et al* (2021)	ActiGraph	Removal of data from a specific time	Choi	Fixed hour requirements	Fixed minimum days	Counts (axis not specified)	Other
Guediri *et al* (2021)	ActiGraph	LFE filter	Not reported	Fixed hour requirements	Fixed minimum days	Not reported	Not reported
Hibbing *et al* (2018)	ActiGraph	Butterworth filter; low pass filter; removal of data from a specific time	Other	Not reported	Not reported	ENMO; SVM; Other	Other (Hibbing *et al* 2018)
Jain *et al* (2021)	ActiGraph	Not reported	Choi	Fixed hour requirements	Fixed minimum days	Counts (axis not specified); Mean of raw acceleration; SD/variance of raw acceleration; Other	Custom cut-points; Machine learning algorithms; Other
Jang *et al* (2020)	ActiGraph; Fitbit	Removal of data from a specific time; other	Zero counts	Fixed hour requirements	Fixed minimum days	Activity counts	Freedson; Other
John *et al* (2019)	ActiGraph; Other	Butterworth filter; Band pass filter; Removal of data above/below threshold; other	Not reported	Not reported	Not reported	Counts (axis not specified); ENMO; Other	Other
Kalisch *et al* (2022)	ActiGraph	LFE filter; removal of data from a specific time	Choi	Not reported	Not reported	Activity counts; Other	Kozey-Keadle; Proprietary
Karas *et al* (2021)	ActiGraph	Not reported	Choi	Percentage-based requirements	Not reported	SVM; Other	Other
Kerr *et al* (2017)	ActiGraph	Removal of data from a specific time; visual inspection	Manual/visual inspection; Choi	Fixed hour requirements	Fixed minimum days	Activity counts; ENMO; Other	Freedson; Matthews; machine learning algorithms; other (Hildebrand)
Lee and Dall (2019)	ActiGraph	Removal of data from a specific time; Other	User-reported data	Not reported	Fixed minimum days	Activity counts	Freedson; Sasaki; Troiano; Other (Santos-Lazano, Hanggi, Morton)
Leonard *et al* (2020)	ActiGraph	Other	Zero counts; User-reported data	Not reported	Not reported	Counts (axis not specified)	Freedson
Loyen *et al* (2016)	ActiGraph	Removal of data above/below threshold	Troiano	Fixed hour requirements	Fixed minimum days	Activity counts	Troiano
Mikkelsen *et al* (2020)	ActiGraph; Fitbit	LFE filter	Choi; user-reported data; other	Fixed hour requirements	Fixed minimum days	Activity counts; other	Proprietary; Sasaki
Montoye *et al* (2024)	ActiGraph	Not reported	Choi; Troiano; Zero counts	Not reported	Not reported	Activity counts	Kozey-Keadle; Sasaki; Other (Montoye *et al* 2020)
Mueller *et al* (2020)	ActiGraph	LFE filter; other	Not reported	Not reported	Fixed minimum days	Activity counts	Freedson; Proprietary; Sasaki; Troiano; other (Williams equation, Crouter 2010)
Nelson *et al* (2019)	ActiGraph	LFE filter	Choi	Not reported	Not reported	Activity counts; other	Freedson; other (Copeland)
Ng *et al* (2019)	ActiGraph	Other	Troiano	Fixed hour requirements	Fixed minimum days	Counts (axis not specified)	Freedson
Nunes *et al* (2024)	ActiGraph	Other	User-reported data	Fixed hour requirements	Weekend inclusion requirement	Counts (axis not specified)	Freedson; Other
O’driscoll *et al* (2021)	ActiGraph; Fitbit	Other	Not reported	Not reported	Not reported	SVM; mean of raw acceleration; percentiles of raw acceleration; SD/variance of raw acceleration; min/max of raw acceleration; dominant frequency of raw acceleration; other	Machine learning algorithms
Poitras *et al* (2020)	ActiGraph	Butterworth filter; band pass filter; removal of data above/below threshold; other	Not reported	Not reported	Not reported	Activity counts	Sasaki
Redenius *et al* (2019)	ActiGraph; Fitbit	Not reported	Choi; user-reported data	Not reported	Not reported	Activity counts	Freedson; proprietary; Sasaki; Troiano
Sheng *et al* (2020)	ActiGraph	LFE filter; high pass filter; removal of data from a specific time	Not reported	Not reported	Not reported	Mean of raw acceleration; SD/variance of raw acceleration; Min/max of raw acceleration; other	Machine learning algorithms
Sillanpää *et al* (2021)	ActiGraph	Removal of data above/below threshold	Not reported	Not reported	Not reported	Activity counts	Sasaki
Stenbäck *et al* (2021)	ActiGraph; Other	Not reported	Not reported	Not reported	Not reported	Counts (axis not specified); SVM; MAD; Other	Freedson; machine learning algorithms; other (Vaha-Ypya)
Thralls *et al* (2019)	ActiGraph	Not reported	Choi	Fixed hour requirements	Fixed minimum days	Activity counts; other	Custom cut-points; machine learning algorithms; other (Copeland, Evenson)
Tomkins-Lane *et al* (2022)	ActiGraph	Not reported	Choi	Fixed hour requirements	Fixed minimum days	Counts (axis not specified)	Freedson; Other
Wang *et al* (2023)	ActiGraph	LFE filter	Troiano; user-reported data	Fixed hour requirements	Fixed minimum days	Activity counts	Sasaki
Weber *et al* (2024)	ActiGraph	Calibration/auto-calibration; consistency checks; Other	GGIR *z*-angle	Fixed hour requirements	Weekend inclusion requirement	ENMO; MAD	Other (Hildebrand, Migueles, Vaha-Ypya)
Webster *et al* (2021)	ActiGraph	LFE filter; Other	Choi	Fixed hour requirements	Fixed minimum days	Activity counts	Other (Aguilar-Farias, Evenson, Koster)
Winfree and Dominick (2018)	ActiGraph; Fitbit	Not reported	Troiano; Zero counts	Fixed hour requirements	Not reported	Activity counts; other	Freedson; Machine learning algorithms
Wing *et al* (2022)	ActiGraph	Visual inspection	Choi	Not reported	Not reported	Activity counts	Proprietary
Xu *et al* (2018)	ActiGraph	Band pass filter	Choi	Fixed hour requirements	Not reported	Activity counts	Freedson
Varma and Watts (2017)	ActiGraph	Other	Choi; user-reported data	Fixed hour requirements	Not reported	Activity counts	Kozey-Keadle; Other
**ActiWatch**							
Hacker *et al* (2017)	ActiWatch	Visual inspection	Zero counts	Fixed hour requirements	Fixed minimum days	Counts (axis not specified)	Other
Kim *et al* (2019)	ActiWatch	Other	Zero counts	Exclusion-based requirements	Not reported	Activity counts	Proprietary
Patel *et al* (2023)	ActiWatch	Not reported	Not reported	Not reported	Fixed minimum days	Counts (axis not specified)	Proprietary
**Axivity**							
Doherty *et al* (2017)	Axivity	Butterworth filter; low pass filter; calibration/auto-calibration; Other	Other	Hourly distribution requirements	Fixed minimum days	ENMO	Other (van Hees 2013 equation)
Liang *et al* (2023)	Axivity	Butterworth filter; low pass filter; calibration/auto-calibration; Other	Other	Hourly distribution requirements	Fixed minimum days	ENMO	Other
**Fitbit**							
Alinia *et al* (2020)	Fitbit; Other	Butterworth filter; low pass filter	Not reported	Not reported	Not reported	Mean of raw acceleration; percentiles of raw acceleration; other	Other
Clevenger *et al* (2019)	Fitbit	Removal of data from a specific time	User-reported data	Not reported	Fixed minimum days	Not reported	Proprietary
Meng *et al* (2020)	Fitbit	Removal of data above/below threshold	Other	Not reported	Fixed minimum days	Not reported	Proprietary
**GENEActiv**							
Dutta *et al* (2018)	GENEActiv	Not reported	Other	Fixed hour requirements	Fixed minimum days	SVM, mean of raw acceleration; SD/variance of raw acceleration; Other	Machine learning algorithms
Jones *et al* (2020)	GENEActiv; other	Removal of data from a specific time	Not reported	Not reported	Not reported	ENMO; mean of raw acceleration; percentiles of raw acceleration; SD/variance of raw acceleration; min/max of raw acceleration; dominant frequency of raw acceleration; other	Machine learning algorithms
Lin *et al* (2023)	GENEActiv	Calibration/auto-calibration	Temperature, light, or other sensors; other	Fixed hour requirements	Not reported	ENMO; other	Other (Migueles)
Montoye *et al* (2018)	GENEActiv	Removal of data from a specific time	Not reported	Not reported	Not reported	Mean of raw acceleration; percentiles of raw acceleration; SD/variance of raw acceleration; min/max of raw acceleration; dominant frequency of raw acceleration; other	Machine learning algorithms
Spulber *et al* (2022)	GENEActiv; ActiWatch	High pass filter; other	Manual/visual inspection	Not reported	Fixed minimum days	Counts (axis not specified); Other	Other
Tsanas *et al* (2020)	GENEActiv	Calibration/auto-calibration; visual inspection; other	Other	Not reported	Fixed minimum days	Other	Other
Vert *et al* (2022)	GENEActiv	Removal of data from a specific time; visual inspection	Manual/visual inspection; GGIR *z*-angle; temperature, light, or other sensors; other	Not reported	Not reported	SD/variance of raw acceleration	Not reported
**Other device or not reported**							
Chuang *et al* (2024)	Other	Band pass filter	Threshold-based methods	Fixed hour requirements	Not reported	Activity counts	Other
Farrahi *et al* (2020)	Other	Not reported	Not reported	Fixed hour requirements	Weekend inclusion requirement	Other	Proprietary; machine learning algorithms
Filippou *et al* (2020)	Other	Butterworth filter	Not reported	Not reported	Not reported	SVM; mean of raw acceleration; SD/variance of raw acceleration; dominant frequency of raw acceleration; other	Machine learning algorithms
Fukuoka *et al* (2018)	Other	Other	Not reported	Fixed hour requirements	Fixed minimum days	Other	Machine learning algorithms; other
Giurgiu *et al* (2019)	Other	High pass filter; low pass filter	Not reported	Fixed hour requirements	Not reported	SVM	Proprietary
Li *et al* (2021)	Other	Not reported	Zero counts	Not reported	Not reported	Activity counts	Proprietary
Liu *et al* (2022)	Other	Butterworth filter; removal of data from a specific time; other	Zero counts	Not reported	Fixed minimum days	Counts (axis not specified)	Other
Nagayoshi *et al* (2019)	Other	High pass filter	Not reported	Not reported	Not reported	Other	Other (Okhawara two-regression, Oshima 2010 equation)
Poli *et al* (2021)	Other	Butterworth filter; high pass filter; low pass filter; other	Not reported	Not reported	Not reported	MAD; mean of raw acceleration; SD/variance of raw acceleration; other	Machine learning algorithms
Rhudy and Mahoney (2018)	Other	Butterworth filter; low pass filter	Not reported	Not reported	Not reported	Other	Other
Sevil *et al* (2020)	Other	Other	Not reported	Not reported	Not reported	Mean of raw acceleration; SD/variance of raw acceleration; min/max of raw acceleration; other	Machine learning algorithms
Spartano *et al* (2023)	Other	Visual inspection	Choi	Fixed hour requirements	Fixed minimum days	Counts (axis not specified)	Other (Colley)
Tanaka *et al* (2023)	Other	High pass filter	Zero counts	Fixed hour requirements	Fixed minimum days	Mean of raw acceleration; Other	Other
Tylcz *et al* (2023)	Other	Low pass filter	Not reported	Not reported	Not reported	Other	Custom cut-points
Wen *et al* (2019)	Other	Not reported	Not reported	Fixed hour requirements	Fixed minimum days	ENMO; Mean of raw acceleration	Other
Xu *et al* (2023)	Other	Calibration/auto-calibration; other	Not reported	Not reported	Fixed minimum days	SVM; Mean of raw acceleration; Percentiles of raw acceleration; SD/variance of raw acceleration; Min/max of raw acceleration	Machine learning algorithms
Batool *et al* (2020)	Not reported	Other	Not reported	Not reported	Not reported	Other	Other
Lee and Gill (2018)	Not reported	Removal of data from a specific time	Zero counts	Percentage-based requirements	Fixed minimum days	Counts (axis not specified)	Proprietary

**Table 9b.** Cleaning and pre-processing techniques of actigraphy data for sleep research.

Study	Accelerometer device	Initial cleaning technique	Non-wear detection algorithm	Daily wear time criteria	Study level wear time inclusion criteria	Activity metrics	Sleep outcomes	Sleep outcome algorithms
ActiGraph								
Barreira *et al* (2018)	ActiGraph	LFE filter	Not reported	Not reported	Fixed minimum days	Activity counts	TST	Sadeh; Custom
Bellettiere *et al* (2019)	ActiGraph	LFE filter	Choi; User-reported data; Other	Not reported	Fixed minimum days	Activity counts	TIB	Other
Chase *et al* (2022)	ActiGraph	Removal of data from a specific time	Other	Not reported	Fixed minimum days	Counts (axis not specified)	WASO; SOL; SE; TST; Other	Cole–Kripke
Lee and Suen (2017)	ActiGraph; ActiWatch	Removal of data above/below threshold	User-reported data	Required during sleep period	Weekend inclusion requirement	Activity counts	WASO; SE; TST	Sadeh; Cole–Kripke; ActiWatch/Actical sleep-wake thresholds; Other
Liu *et al* (2024)	ActiGraph; ActiWatch	Visual inspection	Not reported	Not reported	Fixed minimum days	Activity counts; Counts (axis not specified)	Number of awakenings; WASO; SE; TST; Other	Cole–Kripke; ActiWatch/Actical sleep-wake thresholds
Olsen *et al* (2023)	ActiGraph; Other	Low pass filter; other	Not reported	Required during sleep period	Fixed minimum days	Activity counts; ENMO; MAD; mean of raw acceleration; SD/variance of raw acceleration; min/max of raw acceleration; other	WASO; SOL; SE; TST; other	Custom
Plekhanova *et al* (2023)	ActiGraph; GENEActiv; Axivity	Calibration/auto-calibration; other	GGIR *z*-angle	Not reported	Not reported	Counts (axis not specified); ENMO	WASO; SOL; SE; TST; Other	Sadeh; GGIR
Sansom *et al* (2023)	ActiGraph	Calibration/auto-calibration	GGIR *z*-angle	Fixed hour requirements	Not reported	Counts (axis not specified); ENMO	WASO; SOL; SE; TST; TIB; Other	Tudor-Locke; Cole–Kripke; GGIR
Wing *et al* (2022)	ActiGraph	Visual inspection	Choi	Not reported	Not reported	Activity counts	SE; TST	Cole–Kripke; Other
**ActiWatch**								
Buimer *et al* (2023)	ActiWatch; Other	Removal of data from a specific time; visual inspection	Manual/visual inspection; temperature, light, or other sensors; user-reported data	Not reported	Not reported	Activity counts	WASO; TST; TIB; Other	ActiWatch/Actical sleep-wake thresholds
Gibson and Gander (2019)	ActiWatch	Other	User-reported data; other	Not reported	Not reported	Counts (axis not specified)	Other	ActiWatch/Actical sleep-wake thresholds
Grau *et al* (2022)	ActiWatch; Other	Not reported	User-reported data	Percentage-based requirements	Fixed minimum days	Counts (axis not specified)	WASO; SOL; SE; TST; TIB; Other	ActiWatch/Actical sleep-wake thresholds
Patel *et al* (2023)	ActiWatch	Not reported	Not reported	Not reported	Fixed minimum days	Counts (axis not specified)	Number of awakenings; WASO; SOL; SE; TST; TIB; Other	ActiWatch/Actical sleep-wake thresholds
Rani *et al* (2022)	ActiWatch	Removal of data from a specific time; other	Not reported	Fixed hour requirements	Fixed minimum days	Counts (axis not specified); Other	WASO; TST; Other	ActiWatch/Actical sleep-wake thresholds; Custom
Razjouyan *et al* (2017)	ActiWatch	Band pass filter	Not reported	Not reported	Not reported	Counts (axis not specified)	WASO; SOL; SE; TST	Cole–Kripke; ActiWatch/Actical sleep-wake thresholds
**Axivity**								
Liang *et al* (2023)	Axivity	Butterworth filter; Low pass filter; calibration/auto-calibration; other	Other	Hourly distribution requirements	Fixed minimum days	ENMO	TST	GGIR
**Fitbit**								
Clevenger *et al* (2019)	Fitbit	Removal of data from a specific time	User-reported data	Not reported	Fixed minimum days	Not reported	TST	Proprietary
Liu *et al* (2020)	Fitbit	Not reported	Zero counts	Other	Exclusion-based criteria	Other	TST	Proprietary; other
**GENEActiv**								
Jenkins *et al* (2022)	GENEActiv; ActiWatch	Not reported	Not reported	Not reported	Not reported	Activity counts	WASO; SOL; SE; TST; other	ActiWatch/Actical sleep-wake thresholds
Lavin-Gonzalez *et al* (2020)	GENEActiv	Not reported	Not reported	Exclusion-based requirements	Fixed minimum days	Not reported	TST; other	GGIR; other
Spulber *et al* (2022)	GENEActiv; ActiWatch	High pass filter; other	Manual/visual inspection	Not reported	Fixed minimum days	Counts (axis not specified); other	Other	Other
Tsanas *et al* (2020)	GENEActiv	Calibration/auto-calibration; visual inspection; other	Other	Not reported	Fixed minimum days	Other	Number of awakenings; WASO; TST; other	GGIR; custom
Van Hees *et al* (2018)	GENEActiv; Axivity	Calibration/auto-calibration; other	GGIR *z*-angle	Exclusion-based requirements	Fixed minimum days	ENMO; other	SOL; SE; TST	GGIR
Vert *et al* (2022)	GENEActiv	Removal of data from a specific time; visual inspection	Manual/visual inspection; GGIR *z*-angle; temperature, light, or other sensors; other	Not reported	Not reported	SD/variance of raw acceleration	Did not provide sleep outcomes	Did not provide sleep outcomes
**Other Devices**								
Bailey *et al* (2023)	Other	Not reported	Not reported	Fixed hour requirements	Not reported	Counts (axis not specified)	WASO; SOL; SE; TST; other	Other
Barouni *et al* (2020)	Other	Butterworth filter; band pass filter; other	Temperature, light, or other sensors; Other	Not reported	Not reported	Other	TST	Custom
Chuang *et al* (2024)	Other	Band pass filter	Threshold-based methods	Fixed hour requirements	Not reported	Activity counts	WASO; TST; other	Cole–Kripke
Li *et al* (2021)	Other	Not reported	Zero counts	Not reported	Not reported	Activity counts	Other	Other
Ryser *et al* (2023)	Other	Removal of data above/below threshold	Not reported	Not reported	Not reported	Other	WASO; SOL; SE; TST	Cole–Kripke; custom
Sato *et al* (2023)	Other	Butterworth filter; band pass filter	Not reported	Not reported	Not reported	Other	TST	Other
Wulterkens *et al* (2024)	Other	Not reported	Not reported	Required during sleep period	Nights required	Counts (axis not specified)	Number of awakenings; WASO; SOL; SE; TST; other	Other

Although ActiGraph was the most frequently used device, considerable variation existed even within studies using this brand such as considerable variation in sampling rates, epoch lengths, and wear locations. Similarly, preprocessing techniques varied widely. For example, filtering methods ranged from Butterworth to LFE and band-pass filters, and cleaning approaches included everything from manual inspection to algorithmic thresholding. Notably, 24 studies did not report any cleaning method, underscoring a pervasive gap in clearly reported methods. The major implication of variability in actigraphy cleaning and preprocessing methods is a reduction in cross study comparisons and reproducibility of study findings across research settings. Inconsistent or undocumented approaches make it difficult to replicate analyses, compare outcomes across studies (even when using similar devices or populations), synthesize data for meta-analyses, or draw reliable conclusions about the effects of interventions or exposures due to the added methodological noise (Migueles *et al* 2017).

A few cleaning and pre-processing techniques used in some of the reviewed articles can be used in low resource research settings. With cleaning, visual inspection and fixed non-wear thresholds (e.g., Choi algorithm) can be implemented without proprietary tools or advanced programming knowledge. For pre-processing, the use of open-source algorithms such as GGIR and epoching to standard intervals (e.g., 60 s) provides a replicable foundation that is not resource-intensive and is broadly supported in the literature.

Each set of cut-points (e.g. Freedson, Sasaki, Troiano, Kozey-Keadle and Matthews) were derived from different calibration studies and used different methods and assumptions, which leads to variation in how they are applied. For example, the Freedson cut-points were developed from hip-worn ActiGraph data in young adults using treadmill-based EE and classify moderate activity at 1952–5724 counts/min (Freedson *et al* 1998). Sasaki cut-points also come from treadmill-based studies but focus on vector magnitude data rather than a single axis, shifting the thresholds slightly higher for equivalent intensities (Sasaki *et al* 2011). Troiano cut-points are based on the NHANES and include broader age ranges, offering thresholds like 2020–5999 counts/min for moderate activity (Troiano *et al* 2008). Kozey-Keadle cut-points were developed using overweight, inactive office workers in free-living conditions, with lower sedentary thresholds (<150 counts/min) that reflect minimal movement (Kozey-Keadle *et al* 2011). Matthews cut-points are among the more conservative, classifying moderate activity at ⩾760 counts/min, often leading to higher estimates of time spent in MVPA (Matthew 2005).

Sleep scoring was even less commonly reported, with only 31 of 102 studies including sleep outcomes. Among these, Cole–Kripke and GGIR were the most commonly used algorithms. The small number of studies reporting sleep-related metrics, and the variety of algorithms applied, shows the lack of standardization in actigraphy-based sleep research.

Building upon our synthesis, we provide scenario-driven guidance that connects common study designs to recommended cleaning and pre-processing choices (table 10). For wrist-worn, free-living adult studies, validated non-wear approaches (e.g. Choi or GGIR *z*-angle) paired with diary-based sleep-window alignment and standardized sleep scoring can improve comparability across studies. For hip-based large cohort studies, established wear-time rules (e.g., Troiano-style criteria) and widely used cut-points (e.g., Freedson or Sasaki) are consistent with established surveillance approaches.

**Table 10.** Scenario-based recommendations for selecting actigraphy cleaning and pre-processing methods.

Scenario	Recommended cleaning approach	Recommended pre-processing/algorithm	Rationale/supporting references
Wrist-worn free-living adults	Choi or GGIR *z*-angle non-wear	GGIR or Cole–Kripke for sleep scoring; 60 s epoch	Widely validated; suitable for 24 h wear and sleep–wake detection

Hip-based large cohorts	Troiano 60 min zero-count; fixed ⩾10 h d^−1^ rule	Freedson or Sasaki cut-points	Consistency with NHANES and UK Biobank analyses

Older-adult/clinical studies	Visual inspection or temperature/light sensors	GGIR variance algorithm	Addresses variable compliance and movement patterns

Low-resource/open-source projects	Fixed threshold (⩾90 min zero-count)	GGIR or ACTman	Requires minimal proprietary software; reproducible across devices

Short-epoch/gait analyses	High-frequency (⩾ 80 Hz) sampling; Butterworth filter	Raw ENMO or MAD metrics	Captures brief, high-intensity events with fidelity

In older-adult or clinical samples, where compliance may be intermittent and movement patterns atypical, visual inspection or sensor-assisted detection (e.g. temperature/light when available) may reduce misclassification, and sensitivity analyses are encouraged when alternative thresholds materially change inclusion. In low-resource or open-source settings, fixed non-wear thresholds combined with reproducible approaches (e.g. GGIR or ACTman) can reduce reliance on proprietary software. Finally, for short-epoch or gait-focused analyses, higher sampling rates with appropriate filtering help preserve transient features, and raw-metric outputs (e.g., ENMO/MAD) are preferred when comparability across-devices is required.

Altogether, these findings reveal both the flexibility and the fragmentation of actigraphy data cleaning and pre-processing practices. There is an urgent need for consensus-driven guidelines that promote standardization without limiting adaptability across research settings. Emerging efforts to develop standardized accelerometry reporting recommendations (e.g., Welk *et al* 2019) may help ensure that actigraphy methods are fully described and replicable. Additionally, publicly available code libraries (e.g., GGIR in R, ACTman) and method-sharing platforms can help standardize methods and reduce barriers to high-quality actigraphy analysis, especially in settings with limited computational infrastructure (Kunkels *et al* 2020, Gao *et al* 2021, Suarez *et al* 2024).

### 4.1. Future directions

To help make actigraphy research more consistent, future efforts should focus on creating guidelines that clearly outline essential reporting requirements for cleaning data, preparing it for analysis, and creating outcome measures. These guidelines could be modeled after established frameworks such as STROBE (Cuschieri 2019) or CONSORT (Hopewell *et al* 2025), with adaptations for device-based data. Ideally, these guidelines would be created together by a group of experts from different backgrounds, including researchers, data specialists, software developers, and journal editors, with input from teams working in both well-funded and limited-resource settings. Professional organizations, funding groups, and public health agencies could help lead these efforts. Journals could also play an important role by asking authors to follow clear reporting checklists or by encouraging the use of open-source tools and shared data. Over time, these steps would help others repeat studies more easily, allow for better comparisons between studies, and support larger reviews that combine data from multiple projects.

### 4.2. Limitations

This scoping review has several limitations. First, although our search strategy was comprehensive and guided by a biomedical librarian, we limited inclusion to articles published in English between 2017 and 2024, which may have excluded relevant studies published earlier or in other languages. Second, because we focused on studies that explicitly described actigraphy cleaning and preprocessing methods, articles that used such methods but did not report them in sufficient detail may have been excluded, potentially underestimating usage patterns. Third, due to the scoping nature of the review, we did not assess the quality of study design or risk of bias of the included studies, as our objective was to map the range and variability of methods rather than evaluate study outcomes. Fourth, while we attempted to capture both PA and sleep variables, the relatively small number of studies reporting sleep outcomes limited our ability to summarize findings in that area. Finally, given variation across studies in how methods were reported, including differences in terminology, level of detail, and documentation, as well as differences in device brands and algorithm labels, some categorization decisions (e.g. grouping filters or thresholds) may have introduced minor inconsistencies, despite the use of independent reviewers and group decision-making.

Additionally, while we aimed to categorize data processing methods consistently, some studies provided limited or unclear descriptions of their approaches. This may have led to interpretation or classification differences, especially when distinguishing between cleaning, filtering, and scoring steps. Differences in terminology used by authors (e.g., equating ‘cleaning’ with ‘pre-processing’ or using ‘filtering’ inconsistently) may have influenced how methods were grouped, despite our use of predefined definitions and consensus coding. Moreover, some techniques may be more commonly reported simply due to concentration among high-publishing groups or well-known datasets, which we did not stratify by region or institution. Lastly, we did not report interrater reliability statistics for the full review but calculated agreements during the initial abstract screening phase, where interrater reliability exceeded 85%. All data extraction discrepancies were resolved through team consensus

Still, this scoping review offers a useful overview of how actigraphy data are currently cleaned and pre-processed in research. By covering a wide range of published work, reviewing many studies across different fields, we were able to find common patterns, gaps in reporting, and areas where more consistent methods could help. As is typical for a scoping review, our aim was to describe what is out there, not to evaluate which methods are best. This broad approach can serve as a helpful starting point for researchers looking to improve and standardize actigraphy methods in the future.

## 5. Conclusions

This review contributes to the field by identifying areas where standardization would be most impactful, such as non-wear detection, cleaning transparency, and the selection of PA cut-points. Future reporting guidelines should prioritize transparency in key data processing steps, including cleaning, pre-processing, and algorithm selection. Establishing minimum reporting standards would enable better cross-study comparisons, support secondary analyses, and accelerate reproducibility in PA and sleep research.

## Data Availability

No new data were created or analysed in this study. Reproducibility Checklist available at http://doi.org/10.1088/1361-6579/ae3b96/data1.
